# Functional connectivity heterogeneity and consequences for clinical and cognitive prediction: Stage 2 registered report

**DOI:** 10.1162/IMAG.a.107

**Published:** 2025-08-12

**Authors:** Matthew Mattoni, David V. Smith, Jason Chein, Thomas M. Olino

**Affiliations:** Department of Psychology and Neuroscience, Temple University, Philadelphia, PA, United States

**Keywords:** heterogeneity, ergodicity, resting state, clinical prediction, adolescence

## Abstract

Functional connectivity is frequently used to assess dynamic brain functioning and predict individual differences in behavioral outcomes, such as psychopathology. Inferences from functional connectivity analyses typically rely on group-averaged model statistics. However, heterogeneity between individuals may lead to group-level models that poorly reflect each individual. Poor individual-level precision may limit the ability to make individual-level predictions, which is necessary for key goals such as clinical translation. This registered report examined between-person heterogeneity in resting-state functional connectivity strength patterns by assessing similarity between group- and individual-level connectivity models in the Adolescent Brain Cognitive Development study. Using intraclass correlation coefficients, we found that a group-averaged region-of-interest-based connectivity model was a poor reflection of every individual. In contrast, a group-averaged model of between- and within-network connectivity was a good representation of most individuals. We then examined how individual-level distinctness from the group moderated predictive performance of several clinical and neurocognitive scales. Hypotheses that group-to-individual dissimilarity would worsen behavioral prediction were not supported with primary clinical outcomes. The little psychopathology reported in this sample was a notable limitation. In contrast, lower similarity to the group worsened prediction of performance on the pattern comparison test, providing minor support for hypotheses. Overall, results suggest that region-of-interest-based functional connectivity networks are highly heterogeneous and group-based models are inappropriate for individual-level inferences, but that network-based connectivity is largely similar across individuals. Additionally, we provide minor evidence of the impacts of heterogeneity on prediction that future studies should build on.

## 1 Introduction

Functional connectivity is one of the most common tools to study brain–behavior associations in cognitive neuroscience, particularly for clinical outcomes. Functional connectivity research has led to insights about functional organization of the brain (Yeo et al., 2011), neural correlates of cognitive processes (Gonzalez-Castillo et al., 2015), and risk factors for clinical outcomes (Xia et al., 2018). Resting-state functional connectivity (rsFC) has been of particular interest as it reflects trait-like brain functioning that is largely stable within individuals (Finn et al., 2015) and is easily and reproducibly administered across studies. Despite the promise of rsFC, there has been little success in using rsFC biomarkers to identify those at risk for or diagnosed with psychopathology or predict treatment outcomes. One key factor that may account for the limited clinical translation of rsFC is that inferences are largely based on group-level averages, whereas clinical translation requires individual-specific prediction (Gratton et al., 2020). If rsFC patterns between individuals are substantially heterogeneous, aggregate group-level rsFC networks will not reflect individuals (Molenaar, 2004). This registered report examined group-to-individual similarity of rsFC networks and how prediction may be limited for individuals highly distinct from the group.

The rise of functional connectivity research is tied to recognition that cognitive processes and behaviors result from coordinated neural activity distributed across the brain, rather than from individual regions with unique, specific functions (Pessoa, 2014). Importantly, functional connectivity has several properties that make it primed for the study of individual differences, such as psychopathology. First, rsFC reflects a heritable, trait-like “fingerprint” of neural functioning (Finn et al., 2015; Ge et al., 2017). This property of rsFC networks is critical for investigating neural correlates of trait-like behaviors. Second, while the majority of variation of functional connectivity is explained by trait-level factors, it is also sensitive to state and cognitive demands (Finn & Constable, 2016; Gratton et al., 2018). Thus, functional connectivity can also be used to study changes in clinical symptoms over time (e.g., Strikwerda-Brown et al., 2015). Third, functional connectivity is organized into intrinsic, canonical functional networks across the brain that are replicable across individuals and species (Miranda-Dominguez et al., 2014; Smith et al., 2013; Yeo et al., 2011) and are implicated in distinct cognitive processes (Laird et al., 2011). Network-based analyses are consistent with theoretical perspectives of brain functioning as widely distributed (Pessoa, 2014). Finally, functional connectivity has been shown to have larger associations with behavioral outcomes in multiple domains relative to other neuroimaging measures (Ooi et al., 2022). In sum, rsFC research has high potential to identify neural correlates of clinical functioning. Consequently, its use for clinical study is rapidly increasing (Zhang et al., 2021).

**Fig. 1.**
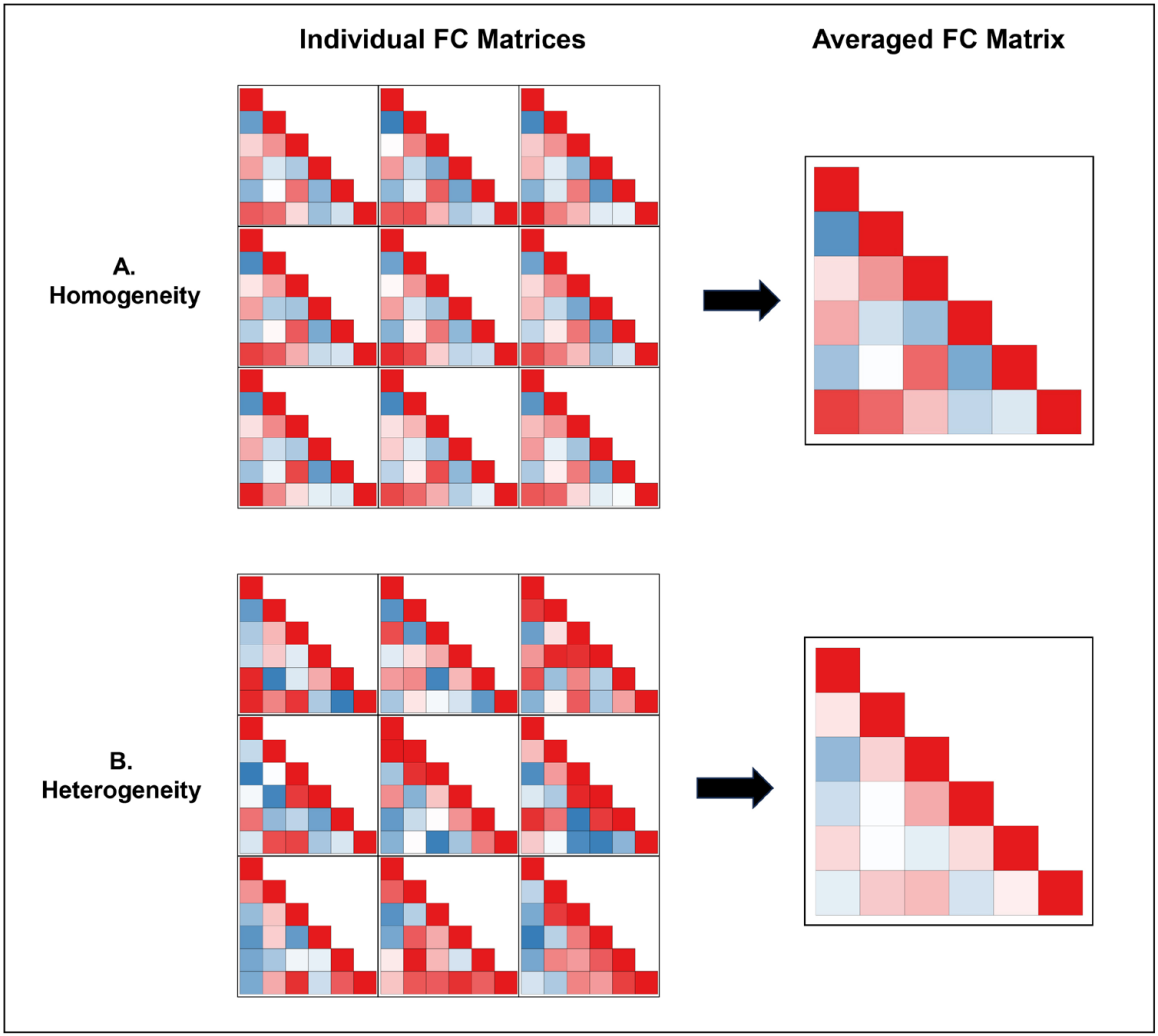
Simulated functional connectivity heterogeneity. *Note.* (A) Homogeneity example. Functional connectivity (FC) correlation matrices were simulated for nine individuals, with each correlation coefficient being within a .30 range across individuals. Due to this similarity across individuals, the group-level rsFC network is representative of each individual. (B) Heterogeneity example. FC matrices were simulated for nine individuals, with all correlations being fully randomized. Due to the heterogeneity between individuals, the group-level matrix does not reflect any single individual.

Despite this promise, clinical translation of functioning connectivity results is exceedingly rare. There are numerous barriers to translation related to limitations of neuroimaging (Dinsdale et al., 2022; Specht, 2020) and the measurement of psychopathology (Tiego et al., 2023). Another key factor that has received less attention is that most rsFC–psychopathology associations are identified at a group level, but clinical prediction requires inferences at the individual level (Gratton et al., 2020). While it may appear reasonable to generalize group-level findings to individuals, it is often the case that group-level findings are not representative of single individuals. Idiographic research has demonstrated that for a process to exhibit group-to-individual generalizability, a property termed *ergodicity*, models of the measured construct need to be homogeneous across individuals (Fisher et al., 2018; Molenaar, 2004). However, as rsFC reliably varies across individuals (Finn et al., 2015; Seitzman et al., 2019) and is high-dimensional in nature, it is unlikely that rsFC networks are homogeneous across individuals. If individuals with distinct rsFC correlation patterns are averaged together, the aggregate network model may reflect few, if any, individuals (Fig. 1). As a consequence, analyses that rely on group aggregation may provide conclusions that are invalid at the individual level (Molenaar, 2004). Limited group-to-individual generalizability that may result from heterogeneity between individuals may be a key factor that has encumbered clinical translation, which requires inferences to be precise at the individual level (Fisher et al., 2018).

Previous work has empirically demonstrated that rsFC measures systematically differ across individuals and that group-level models may not reflect individuals. Finn et al. (2015) found that rsFC correlation matrices are reliably matched to individuals with high accuracy, suggesting both distinctness across individuals and stability within individuals. More recently, precision imaging studies have captured robust differences in the spatial organization of functional networks between individuals that are obscured in the group average (Gordon et al., 2017; Laumann et al., 2015). Precision imaging studies have also shown that rsFC networks of nearly every individual differ from group-level organization (Seitzman et al., 2019). Similar results have been found in task-based studies, including findings of stable individual-level task activation maps differing from the group (Miller et al., 2009; Nakuci et al., 2023) and aggregated effective connectivity networks poorly reflecting most constituent individuals (Mattoni et al., 2023).

Despite findings that aggregated functional connectivity networks do not accurately reflect individuals, no study to our knowledge has quantified group-to-individual similarity of rsFC networks or examined the potential impact of this heterogeneity on clinical or behavioral prediction. This is a critical gap in the literature, as many studies of neural correlates of psychopathology and other key phenotypes rely on group-level rsFC networks. This study evaluated similarity of group and individual rsFC networks and examined how the prediction of clinical outcomes may differ for individuals who are dissimilar from the group. Although there are numerous ways rsFC networks can differ between individuals (Gordon & Nelson, 2021), we specifically focused on variation in connectivity strength as this is the most frequently used measure in studies of individual differences. We conducted these analyses using both ROI and intrinsic network-based connectivity, as both approaches are common in the field and a network-based approach substantially reduces the number of dimensions. We registered hypotheses that clinical prediction models trained on group-level rsFC features will perform worse for individuals increasingly dissimilar from the group. The findings of group-to-individual generalizability of rsFC networks and behavioral prediction are relevant for any research reliant on aggregate rsFC networks, but are particularly important for the study of psychopathology as goals of clinical translation ultimately require inferences to individuals.

## 2 Methods

This study is a secondary data stage 2 registered report. The stage 1 report, approved following peer-reviewed by Peer Community In Registered Reports, can be found on OSF (https://osf.io/4w8hb). Deviations from the stage 1 report are described below and further indicated in footnotes. Code to reproduce all analyses using data from the Adolescent Brain Cognitive Development study Community Collection can also be found on OSF: https://osf.io/kdx65.

### 2.1 Deviations

There are three deviations from the stage 1 report. First, the stage 1 report identified “nihtbx_flanker_rawscore” and “nih_pattern_rawscore” as the designated variables for the secondary outcomes. Upon accessing the data, it became evident these variables were inappropriate for analyses. Nearly every participant had the ceiling score (e.g., 20 for Flanker), indicating that this variable reflects trials completed, not the intended NIH Toolbox scoring system accounting for accuracy and reaction time (Luciana et al., 2018). We instead used “uncorrected” scores, similar to previous ABCD analyses (e.g., Marek et al., 2022). Second, the stage 1 report indicated that tSnR would be calculated in the whole sample for exclusions, rather than the training set. Using the whole sample would inappropriately break independence of training and testing sets, an important assumption for prediction (Scheinost et al., 2019), so ROI feature selection was completed in the training set independently and subsequently applied to the testing set. However, post hoc analyses revealed an identical set of ROIs were selected with either approach, rendering the deviation practically meaningless. Third, the stage 1 report indicated an inferential preference for network-based models rather than ROI-based models based on an incorrect assumption that ICC necessarily increases with the number of dimensions. As ICC does not have a defined relationship with the number of dimensions, we instead interpret both sets of results separately but equally.

### 2.2 Participants

Data came from the Adolescent Brain Cognitive Development (ABCD) study, a 21-site study of over 11,000 children focused on examining how neurobiological and environmental changes may influence youth health and functioning over time (Casey et al., 2018). The ABCD study team obtained informed consent from all participants included in this study (Garavan et al., 2018). The ABCD study provides the large sample size of high-quality fMRI data necessary for reliable associations (Marek et al., 2022). Full details of ABCD study recruitment and sample characteristics are available elsewhere (Casey et al., 2018; Garavan et al., 2018). The demographically diverse, population-based sample was recruited to mirror demographic characteristics from the American Community Survey (ACS) with regard to race, ethnicity, sex, SES, and urban/rural residency. Recruitment was primarily school based, with approximately 10% of the sample recruited through additional means (e.g., mailing lists, twin identification from birth registries). This study examined data from the second wave of the ABCD study, which includes 11–12-year-old early adolescents. We examined the second wave due to the potential of older children having less head motion (Satterthwaite et al., 2012) and greater variability in clinical symptomatology. Initial quality control was assessed by the ABCD study team (Casey et al., 2018). We included participants who passed ABCD resting-state quality control measures and had at least 10 minutes of resting-state data with less than .10 mm framewise displacement (FD). Finally, we split the sample into equal training and testing sets using assignments from the ABCD Community Collection (ABCC). The ABCC is a curated version of ABCD data with preprocessed fMRI data and participant assignments into separate training and testing sets, such that the subsets are highly similar across nine sociodemographic variables and account for familial nesting (Feczko et al., 2021). At baseline, each set contained 5,786 participants before exclusion based on fMRI QC or missing data.

### 2.3 Measures

#### 2.3.1 fMRI measures

We used whole brain resting-state BOLD time series extracted from the ABCC (Feczko et al., 2021). The ABCC preprocessed data based on a pipeline used in the Human Connectome Project (Glasser et al., 2013). Briefly, preprocessing steps completed by the ABCC team include anatomical normalization, cortical surface reconstruction, registration of data to standardized surface space, functional data distortion correction, registration to template surfaces, and normalization. Further details can be found in Feczko et al. (2021). Postprocessing steps also completed by the ABCC team are from the DCAN-Labs processing pipeline (https://github.com/ABCD-STUDY/nda-abcd-collection-3165/blob/main/docs/pipelines.md). A respiratory filter was used to improve FD estimates and censor frames with a filtered-FD greater than .3 mm (Fair et al., 2020). Data were further processed by demeaning, detrending, and denoising with global, ventricular, and white matter signal regressors, as well as translational and rotational movement measures. Censored frames were then interpolated and the time series were passed through a band-pass filter of 0.008 Hz < f < 0.1 Hz. Data were then parcellated based on several atlases. We extracted time series from the Gordon atlas (Gordon et al., 2016), which consists of 333 regions of interest (ROIs) with a priori assignments to intrinsic functional connectivity networks as well as 19 additional subcortical ROIs from the FreeSurfer atlas (352 total ROIs). We concatenated time series across the four runs and censored frames with filtered-FD > .10 to reach the final time series for each individual.

#### 2.3.2 Outcome measures

Clinical outcomes were internalizing and externalizing scale scores from each of the parent-reported Childhood Behavioral Checklist (CBCL; Achenbach, 2009) and youth-reported Brief Problem Monitor Scale (BPMS; Achenbach, 2009). Each measure was examined separately. The CBCL internalizing scale (cbcl_scr_syn_internal_r) contains items from the withdrawn, somatic complaints, and anxiety/depressed problems subscales, and the externalizing scale (cbcl_scr_syn_external_r) contains items from the delinquent and aggressive behaviors subscales. The BPMS internalizing scale (bpm_y_scr_internal_r) contains items related to anxiety and depression symptoms, and the externalizing scale (bpm_y_scr_external_r) contains items related to delinquent and aggressive behaviors. As secondary outcomes, we also examined flanker task (nihtbx_flanker_uncorrected) and pattern comparison (nihtbx_pattern_uncorrected) performance (see Sensitivity Analyses) as measures of inhibitory control and processing speed, respectively.^*^ Distributions and summary statistics of outcome measures are presented in the Supplementary Data. There was little psychopathology reported overall in the sample.

### 2.4 Analysis plan

See Table 1 for a summary of primary research questions (RQs) and Supplementary Figure S1 for an overview of the analytic plan. Our overall goal was to examine how group-level rsFC networks (RQ1) and brain–behavior associations (RQ2) apply to individuals. We examined these research questions using ROI-based rsFC as well as intrinsic network connectivity. Analyses steps were repeated for both rsFC approaches. As this study is exploratory, the registered report sets a principled, a priori plan to address the research questions and guide the analytical options. All analyses were completed in R version 4.2.2, specific packages are described below.

**Table 1.** Study design summary.

Research question	Hypothesis	Analysis plan	Interpretations
*ROI-Based rsFC:*			
1A. Group-to-Individual Similarity	Exploratory	Calculate ICC between training group and each individual’s ROI-ROI rsFC. Explore distribution.	We will use common benchmarks of ICC < .50 as poor group-to-individual similarity, .50 > ICC > 0.75 as moderate similarity, ICC > .75 as good similarity, and ICC > .90 as excellent similarity. Much of the distribution of individual ICC values being near excellent would reflect a relatively homogeneous sample. The center of the distribution being in the moderate range or lower would reflect a heterogeneous sample.The similarity distribution in the training sample will reflect group-to-individual generalizability. The distribution in the test sample will reflect the ability of the group-level network to generalize to unseen data, a requirement for population generalizability.
2A. Effect on individual-level clinical inferences	**Null (N):** Outcome prediction model performance will not differ based on rsFC similarity to the group. **Hypothesis (H):** Group similarity will moderate model performance, such that higher group similarity values will be associated with *stronger* relationships between the predicted and observed outcome values. **Alternative (A):** Group similarity will moderate model performance, such that higher group similarity values will be associated with *weaker* relationships between the predicted and observed outcome values.	Test moderation effect of ICC on the relationship between rsFC model-predicted outcomes and observed outcomes. Significance will be assessed at α = .05.Performance in the train and test subsets will be examined separately and interpreted as explanatory and predictive, respectively.	**N:** A non-significant interaction would suggest that rsFC distinctness from the group does not impact prediction accuracy of outcomes from rsFC features. **H:** A significant interaction with a positive coefficient for model performance by ICC would suggest that prediction of brain–behavior associations is weaker for individuals who are less similar to the group model. This would suggest that rsFC heterogeneity limits clinical prediction. **A:** A significant interaction with a negative coefficient for model performance by ICC would suggest that prediction of brain–behavior associations is stronger for individuals who are less similar to the group model. This would suggest that clinical prediction accuracy is in part explained by deviating from the group model.
*Network-Based rsFC*			
1B. Group-to-Individual Similarity	Exploratory	Calculate ICC between training group and each individual’s between- and within-network rsFC. Explore distribution.	We will use the same inferential framework as RQ1.Substantially different ICC values relative to ROI-based rsFC may suggest ICC values are influenced by the large number of features. In this case, we will conduct several sensitivity analyses (see Sensitivity Analyses).
2B. Effect of individual-level clinical inferences	**Null (N):** Outcome prediction model performance will not differ based on rsFC similarity to the group. **Hypothesis (H):** Group similarity will moderate model performance, such that higher group similarity values will be associated with *stronger* relationships between the predicted and observed outcome values. **Alternative (A):** Group similarity will moderate model performance, such that higher group similarity values will be associated with *weaker* relationships between the predicted and observed outcome values.	Test moderation effect of ICC on the relationship between model-predicted outcomes and observed outcomes. Significance will be assessed at α = .05.Performance in the train and test subsets will be examined separately and interpreted as explanatory and predictive, respectively.	We will use the same inferential framework as RQ2. Substantive interpretations will be separate for the ROI- and network-based models and overall conclusions will focus on the ensemble results.

*Sampling Plan*: The sampling plan for each research question will consist of all available data from the ABCD study that pass inclusion criteria (See Participants). *Rationale for Hypothesis Test*: Due to the novelty of the research question, we do not hypothesize effect size and rely on traditional significance test at α = .05. However, as rsFC values have typically explained a small amount of variance in clinical outcomes in the ABCD dataset, we expect any differences in prediction accuracy by ICC to be small.

#### 2.4.1 ROI-based rsFC

We first characterized ROI-based connectivity heterogeneity by testing how well a group-average functional connectivity network represents individuals. We reduced the number of features to limit the large number of rsFC paths (352 ROIs = 61,776 correlations), focus on theoretically relevant paths, and mitigate the influence of noisy ROIs. We first limited ROIs to those with a priori assignments to various association networks, rather than primary motor or sensory networks. These included the default mode, salience, frontoparietal, cingulo-parietal, retrosplenial-temporal, cingulo-opercular, ventral attention, and dorsal attention networks as well as the 19 FreeSurfer subcortical ROIs (196 ROIs, 19,110 correlations). A list of all Gordon atlas ROIs is available on OSF: https://osf.io/yk7cp/?view_only=64b20df7958d4dd6b1eecf6a208b92b0). These association networks have more intersubject variance, are more relevant to the study of psychopathology (Chen et al., 2022; Finn et al., 2015), and typically have higher retest reliability relative to paths in other networks (Noble et al., 2019). However, reliability is typically still low, indicating a limitation of rsFC for prediction. To reduce the possibility that gross variability in signal (i.e., noise) impacts model estimation and inferences, we calculated the mean temporal signal-to-noise ratio (tSnR) for the training set by dividing each subject’s mean signal by the standard deviation for each individual ROI.^†^ For the primary analysis, we excluded ROIs with a tSnR 2 standard deviations below the mean to focus on regions with better signal. We repeated analyses relying on a threshold of 1 standard deviation below the mean as sensitivity analysis.

Using the reduced set of ROIs, we estimated Pearson correlation matrices (pairwise correlation of time series for all remaining ROIs) for each individual in the training set. No covariates were included in correlation estimations. To further limit the features to those with interindividual variance (necessary for behavioral prediction), we selected the features with the highest 10% of variance estimates between individuals, providing the final list of rsFC features. We then Fisher z-transformed coefficients (*fisherz* in “psych” R package) and estimated the group average rsFC network by taking the mean Fisher z-coefficient for each path across participants.

#### 2.4.2 Network-based rsFC

In addition to the ROI-based approach, we examined group-to-individual similarity of intrinsic network-based rsFC. A network-based approach provides increased interpretability for clinical prediction due to the substantially fewer number of features, and may provide distinct conclusions from an ROI-based approach. Moreover, the large number of features in the ROI-based approach may inflate ICC estimates. We included the same association networks as used in the ROI-based approach (default mode, salience, frontoparietal, cingulo-parietal, retrosplenial-temporal, cingulo-opercular, ventral attention, and dorsal attention) and excluded sensorimotor networks and subcortical regions. Unlike the ROI-based approach, we did not perform further feature reduction, as both the consequences of ROI-specific noise and the total number of features are already mitigated by aggregating across networks. Using a priori ROI assignments to intrinsic functional networks, we calculated within-network connectivity (average correlation between all ROIs in a network) for each network and between-network connectivity (average correlation between all ROIs in two separate networks) for each pair of networks. This resulted in an 8 x 8 connectivity matrix with the diagonal representing within-network connectivity (36 distinct correlations).

#### 2.4.3 Group-to-individual similarity

We estimated intraclass correlation coefficients (ICCs) between the group-level rsFC matrix and each individual-level rsFC matrix as an index of group-to-individual rsFC similarity. We used ICCs to consider both rank-order and mean-level similarity of rsFC. ICC was estimated using the “IRR” package in R, using a two-way random effects model with absolute agreement and single unit rater (Shrout & Fleiss, 1979). Similarity in the training set reflects how well a group-level network represents each constituent individual. The distribution of ICCs will show the range of (dis)similarity between the group and individual rsFC networks. We then calculated group-to-individual similarity in the testing set, still using the group-level model from the training set. Similarity in the testing set reflects the ability for a large group average to generalize to unseen data, an important property for population-level inferences. ICC was estimated separately for ROI-based rsFC and network-based rsFC. The ICC distributions were the primary outcomes used as estimates of rsFC network similarity. The stage 1 report noted that substantive inferences would be more informed by the network-based approach, incorrectly suggesting that ICC estimates are inflated by the number of dimensions. As this is not an accurate depiction of ICC, we do not maintain an inferential bias toward ROI-based or network-based models and instead interpret them separately.^‡^

#### 2.4.4 Clinical prediction by group similarity

We examined how group-to-individual similarity affects the ability to identify clinical brain–behavior associations that apply to each individual. Briefly, we trained an elastic net nested cross-validation model on the training set to predict clinical and executive functioning outcomes from rsFC matrices. We used the elastic net model to estimate predicted values for each outcome variable for all individuals in the training and testing sets, separately. Finally, we evaluated how model performance differs by group similarity by testing the interaction between ICC and predicted value (from elastic net model) on the observed value. Models were estimated separately for ROI-based rsFC and network-based rsFC, the 4 clinical measures (primary outcomes), and the 2 executive functioning measures (secondary outcomes), totaling 12 models. Participants with missing data for an outcome were excluded from that model.

Elastic net models were trained using 10-fold cross-validation using the *cv.glmet* function in the “glmnet” package (Friedman et al., 2010) in R. We selected the best performing model (combination of α and λ parameters) by the minimum mean standard error. The best performing elastic net model was used to estimate predicted values for each outcome, for each individual. We examined the effect of group similarity on behavioral prediction by moderation in linear regression. The main effect of predicted outcome on observed outcome reflects the overall model performance (similar to estimating the correlation between predicted and observed outcomes as a metric of model performance, e.g., Dhamala et al., 2023), but with a coefficient of 1 indicating optimal prediction. The main effect of group similarity on observed outcome reflects the effect of (dis)similarity in individuals’ rsFC features from the group average on psychopathology outcomes. The interaction effect was the primary focus and reflects how model performance is moderated by rsFC group similarity. We assessed significance of each effect at α = .05 and further examined regions of significance of interactions in Johnson–Neyman plots.

We examined power using the *InteractionPoweR* package in R (Baranger et al., 2023). Various a priori power calculations that assumed training and testing sample sizes of 2,500 can be found on OSF at https://osf.io/93mau. Additional power calculations with the true analytical sample size of 1,165 can be found at https://osf.io/z67md. As one example, with assumptions of r = .10 between all model variables and retest reliability of .75, we had 80% power to detect an interaction effect as small as r = 0.13.

Due to the number of similar analyses, our overall inferential focus was on the broader pattern of results across the various rsFC matrices and outcomes. As child- and parent-reported measures contain information from different contexts and factors (De Los Reyes et al., 2015), we interpret informant results separately and do not prioritize results from any single informant. Similarly, neither the ROI-based nor network-based approach was preferred for the moderation of clinical prediction as they are assessing distinct aspects of network heterogeneity. Associations in the testing set were emphasized for predictive inferences.

#### 2.4.5 Sensitivity analyses

We planned several sensitivity analyses to examine the consequences of analytical choices and to clarify the implications of results.

##### 2.4.5.1 Feature reduction

It is possible that ICC values may be substantially different in the ROI-based approach relative to the network-based approach due to the number of features, rather than true differences in group-model representativeness. For instance, we expected that ICC may increase with the number of dimensions. To examine this, we repeated ICC estimation after restricting FC features to those with top 50%, 25%, 5%, and 1% of interindividual variance (compared with 10% in main analyses). Additionally, reducing features to the paths with the most interindividual variance may bias the feature set to noisier regions. To examine the impact of feature count and noise within ROIs, we used a stricter tSnR threshold of 1 standard deviation below the mean. This stricter threshold simultaneously reduces the number of features and noise.

##### 2.4.5.2 Outcome measures

Internalizing and externalizing symptoms were the primary outcomes. However, effect sizes between neuroimaging measures and clinical outcomes are often very small (Marek et al., 2022), potentially limiting the ability to detect effects of group similarity on prediction. We also examined measures of executive functioning that have stronger association with rsFC (Marek et al., 2022). We specifically examined the NIH Toolbox’s Flanker test as a measure of cognitive control (Eriksen & Eriksen, 1974) and the NIH Toolbox’s Pattern Comparison test as a measure of processing speed (Carlozzi et al., 2014).

##### 2.4.5.3 Covariates

We examined how age, sex, scanning site, and mean framewise displacement are associated with group similarity in separate linear models. We also estimate supplemental moderated regressions that control for confounds that were found to be significant. This sensitivity analysis informs whether the potential moderation of model performance by group similarity is better explained by confounding variables. Significance was defined at α = .01 after Bonferroni correction. We used a stricter significance threshold due to concerns of introducing bias by partialing variance (Hoyle et al., 2023).

## 3 Results

### 3.1 Sample description

After the preregistered exclusion criteria, the final sample was N = 2,330, with 1,165 participants in each of the training and testing sets. Full demographic information on the final sample is presented in the Supplementary Data (Table S1). Briefly, the mean framewise displacement was .08 mm in both the training and testing sets after exclusions and the subsets remained highly similar to each other in demographic factors, psychopathology, and neurocognitive performance after exclusions. Relative to the full population-based ABCD sample at baseline (see Supplementary Data), the analytical sample here (2 years older and exclusion criteria focused on minimizing scanner motion) had slightly more participants who were assigned female at birth, substantially more individuals identified as White, slightly more individuals of higher income, slightly lower youth-reported psychopathology symptoms, and substantially higher neurocognitive performance.

After selecting ROIs assigned to targeted networks, excluding ROIs with a tSnR 2 standard deviations below the mean, and selecting ROI–ROI correlations with the top 10% of interindividual variance, there were 1,721 connectivity features. These features included 1,070 ROIs in the default mode network, 743 in the frontoparietal network, 739 in the dorsal attention network, 450 in the ventral attention network, 435 in the cingulo-opercular network, 3 in the retrosplenial-temporal network, 1 in the cingulo-parietal network, and 1 in the salience network. No subcortical regions were included.

### 3.2 Group-average versus individual-level connectivity networks

**Fig. 2.**
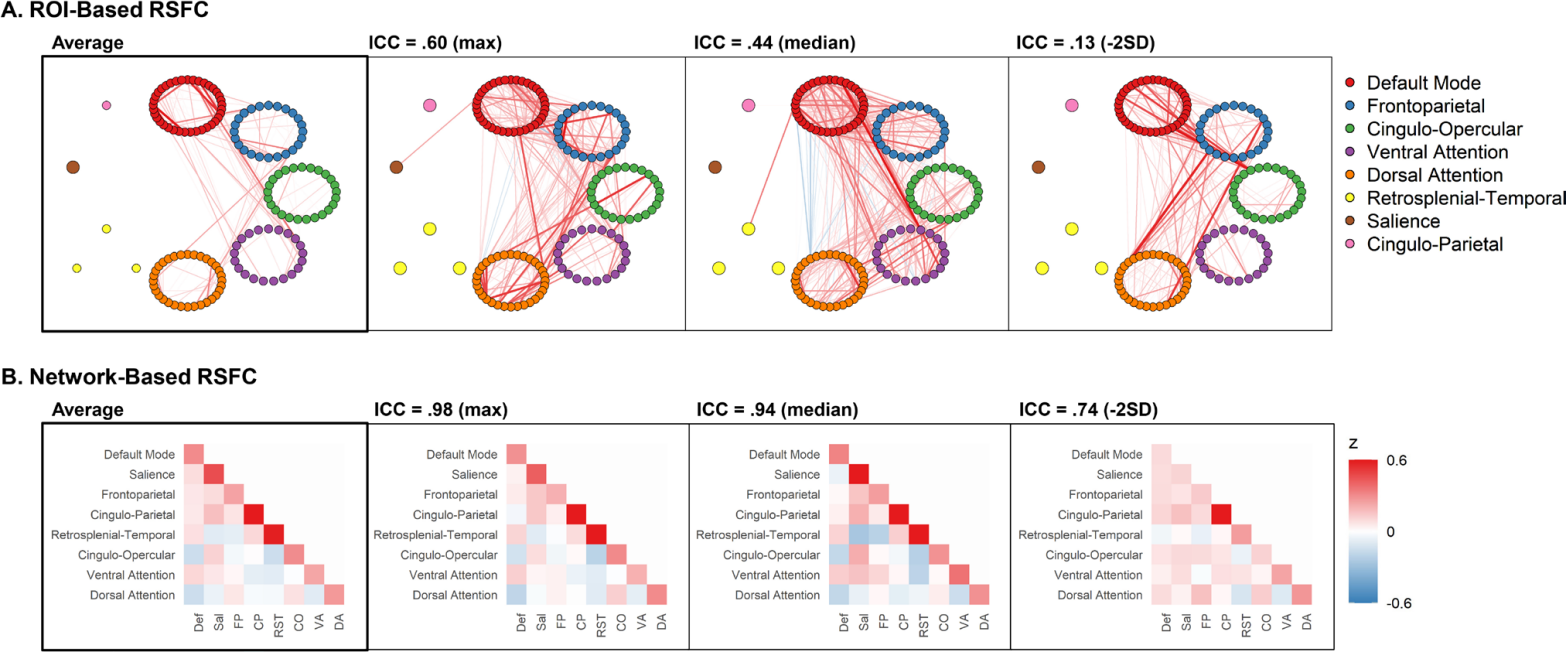
Group-average and individual-level rsFC. *Note.* For each row, the first plot on the left represents the group average. The second, third, and fourth plots display the individual with the highest, median, and -2 standard deviation group-to-individual similarity ICC values, respectively. (A) Network diagrams of ROI-based connectivity from the training set. Dots represent ROIs, with colors indicating network assignment. Networks with more ROIs are clustered into a ring to visualize intra-network functional connectivity. Edges (paths) represent Fisher z-transformed correlation values; red edges are positive and blue are negative. Only z-transformed correlations greater than or equal to |.40| are shown. (B) Correlation matrices (Fisher z-transformed) of network-based connectivity from the training set. The within-network connectivity for the cingulo-parietal network was higher than .60 for each matrix, but capped at .60 for visualization purposes of other elements.

**Fig. 3.**
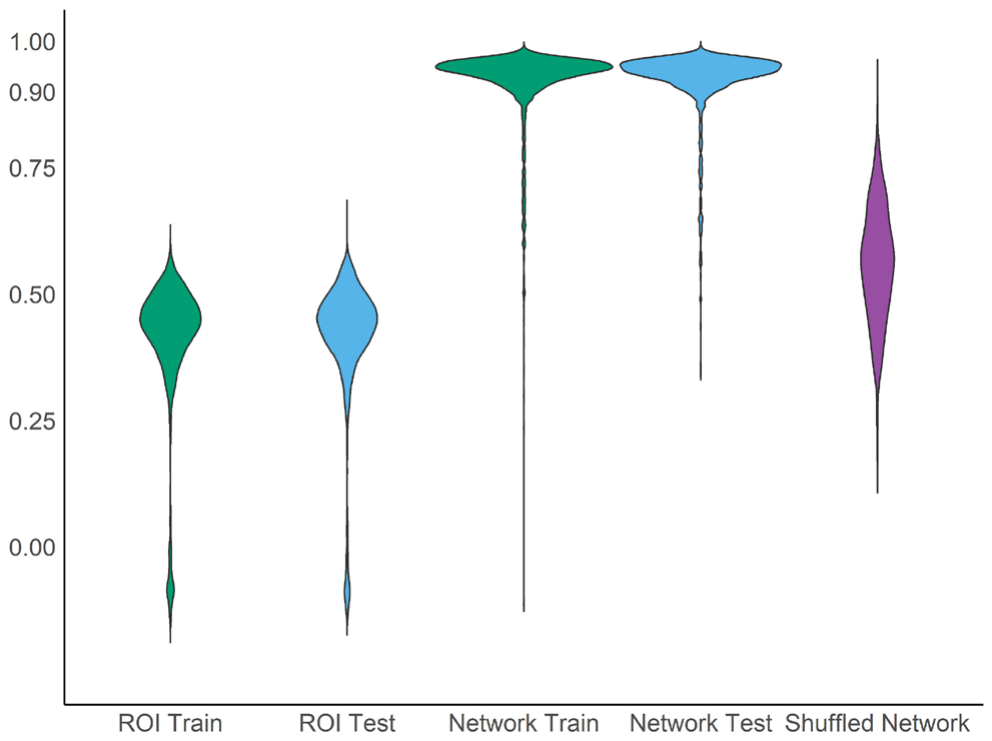
Distributions of group-to-individual similarity ICC. *Note.* Violin plots of ICC distributions. ICC reflects the intraclass correlation between the RSFC network of each individual and the group average, reflecting group similarity. The shuffled network violin (purple) reflects the mean ICC across the sample when network assignments were randomly shuffled across ROIs, breaking true network structure (500 iterations). The substantially lower shuffled ICC distribution suggests the high network-based ICC is not due to data aggregation.

We first estimated ROI-based rsFC using preregistered ROIs and canonical network-based rsFC by averaging correlations between ROIs between and within preregistered networks. The average ROI-based rsFC network for the training set is presented in Figure 2A and the average network-based rsFC network for the training set is presented in Figure 2B. We examined the similarity between the training set average rsFC network and each individual’s network through ICCs. ICC distributions were negatively skewed and are presented in Figure 3.

#### 3.2.1 Exploratory Hypothesis 1A

*Explore distribution of ROI-based group-to-individual ICC estimates, using benchmarks of poor ICC* *<* *.50, moderate 0.50* *<* *ICC* *<* *0.75, and good ICC* *>* *.90.*

ROI-based rsFC showed poor ICC for every individual, with a relatively narrow distribution centered at 0.41 (SD = 0.14), suggesting substantial heterogeneity. The ICC distribution in the testing set (compared with the training set’s group average) was similarly poor (M = 0.41, SD = 0.13). Comparisons between the group average network and three select individuals are presented in Figure 2A. With poor ICC similarity values, even the most similar individual (max ICC = 0.60) does not reflect the group average.

#### 3.2.2 Exploratory Hypothesis 1B

*Explore distribution of network-based group-to-individual ICC estimates, using benchmarks of poor ICC* *<* *.50, moderate 0.50* *<* *ICC* *<* *0.75, and good ICC* *>* *.90.* Network-based rsFC showed strong ICC, with a narrow distribution centered at 0.92 (SD = 0.09), suggesting homogeneity. The distribution in the testing set was similarly excellent (M = 0.93, SD = 0.07). Comparisons between the group average and three select individuals are presented in Figure 2B. The presented individuals with ICC values greater than .90 are largely homogeneous and are largely reflected by the group average. Some notable differences are observable in the presented individual with ICC of .74 (e.g., correlation patterns with the dorsal attention network).

#### 3.2.3 Post hoc analysis

Group-to-individual ICC values were substantially higher in network-based rsFC models relative to ROI-based rsFC. We completed non-preregistered exploratory analyses to examine whether this difference was due to a true homogeneous network structure or an artifact from averaging and feature reduction. Across 500 iterations, we randomly shuffled network assignments for each ROI before estimating between- and within-network rsFC and corresponding ICCs (Fig. 3). Across the 500 iterations, the mean iteration-wise sample ICC average was 0.56 (SD = 0.24 across iterations) and the standard deviation of iteration-wise ICC averages was 0.11. No shuffled iteration had a mean ICC as high as the mean with the true network structure of 0.92 (max = 0.88). This negative shift in group-to-individual ICCs suggests that it is the true network structure, not aggregation across ROIs, leading to high group similarity.

### 3.3 Prediction of behavioral outcomes and ICC moderation

We then estimated elastic net models predicting child- and parent-reported internalizing and externalizing symptoms from ROI- and network-based rsFC, separately. Performance on the Flanker and Pattern Comparison was examined as secondary outcomes. Distributions and scatterplots of observed outcome scores by ICC and by predicted scores are presented in the Supplementary Data (Supplementary Figs. S2-S4). Predictive performance of each model in the training and testing sets is shown in Table 2 for ROI-based rsFC and Table 3 for network-based rsFC. Consistent with prior work in the baseline sample (Marek et al., 2022), predictive performance was overall low (correlation between predicted and observed testing set scores ranged from r = 0 to 0.14). No features were selected for prediction of CBCL internalizing. Neither ROI-based nor network-based models were systemically better at predicting outcomes across the different measures. Additionally, models were overfit to the training set, as shown by substantial decreases in model performance from training to testing sets.

**Table 2.** ROI-based prediction of behavioral outcomes and moderation by ICC.

	CBCL Int	CBCL-Ext	BPM-Int	BPM-Ext	Flanker	Pattern
Training Set						
Elastic Net Prediction						
r	0	0.24	0.41	0.19	0.47	0.48
Selected Features	0	351	587	273	635	696
Moderation Model						
Intercept	-	3.35 (0.14)***	1.60 (0.06)***	1.99 (0.06)***	101.03 (0.19)***	105.24 (.40)***
Predicted Score	-	4.06 (0.49)***	5.35 (0.35)***	4.25 (0.64)***	4.13 (0.24)***	3.76 (0.21)***
ICC	-	-0.24 (1.02)	0.98 (0.43)*	0.52 (0.76)	0.56 (1.36)	-3.43 (2.82)
Predicted Score*ICC	-	-1.77 (3.30)	-4.66 (3.31)	3.76 (3.25)	1.12 (1.91)	3.37 (1.07)**
Testing Set						
Elastic Net Prediction						
r	0	0.04	0.06	0.03	0.14	0.06
Moderation Model						
Intercept	-	3.56 (0.14)***	1.77 (0.07)***	2.06 (0.07)***	101.00 (0.21)***	104.97 (.44)***
Predicted Score	-	0.59 (0.54)	0.86 (0.45)	0.89 (0.72)	1.39 (0.31)***	0.55 (0.27)*
ICC	-	-1.56 (1.18)	-0.06 (0.59)	0.46 (0.82)	-0.20 (1.63)	0.51 (3.48)
Predicted Score*ICC	-	-0.48 (5.22)	2.99 (5.31)	-0.45 (3.61)	3.10 (3.09)	5.91 (1.99)**

*Note.* *p < .05, **p < .01, ***p < .001. Performance of elastic net models with ROI-based connectivity features; r is the correlation between elastic net-predicted score and observed score (i.e., model performance). Selected features reflect the number of features retained by elastic net. Regression coefficients are reported for the moderation model predicting observed scores from the interaction of predicted scores and ICC. A regression coefficient of 1 for a main effect or a conditional effect would reflect optimal prediction. There were no selected features for CBCL internalizing scores.

**Table 3.** Network-based prediction of behavioral outcomes and moderation by ICC.

	CBCL Int	CBCL-Ext	BPM-Int	BPM-Ext	Flanker	Pattern
Training Set						
Elastic Net Prediction						
r	0	0.09	0.18	0.13	0.19	0.21
Selected Features	0	15	29	32	32	34
Moderation Model						
Intercept	-	3.49 (0.16)***	1.62 (0.06)***	1.97 (0.06)***	101.02 (0.21)***	105.34 (0.45)***
Predicted Score	-	3.44 (1.23)**	2.41 (0.40)***	2.19 (0.49)***	2.95 (0.48)***	1.92 (0.28)***
ICC	-	-5.82 (3.92)	0.02 (0.70)	0.11 (1.48)	-1.27 (2.59)	-2.06 (5.11)
Predicted Score*ICC	-	20.55 (10.66)	8.16 (6.30)	3.33 (4.99)	0.34 (5.30)	-0.89 (3.96)
Testing Set						
Elastic Net Prediction						
r	0	0.02	0.09	0.05	0.04	0.08
Moderation Model						
Intercept	-	3.62 (0.16)***	1.77 (0.07)***	2.10 (0.06)***	100.99 (0.21)***	105.21 (0.44)***
Predicted Outcome	-	-0.33 (1.29)	1.28 (0.42)**	1.02 (0.52)	0.54 (0.48)	0.77 (0.27)**
ICC	-	-6.45 (4.59)	0.51 (0.98)	-2.06 (1.62)	-1.55 (3.04)	-8.66 (6.70)
Predicted Score*ICC	-	9.15 (15.18)	7.12 (7.46)	8.83 (4.94)	-7.13 (7.34)	-0.06 (3.56)

*Note.* *p < .05, **p < .01, ***p < .001. Performance of elastic net model with network-based connectivity features; r is the correlation between elastic net-predicted score and observed score (i.e., model performance). Selected features reflect the number of features retained by elastic net. Regression coefficients are reported for the moderation model predicting observed scores from the interaction of predicted scores and ICC. A regression coefficient of 1 for a main effect or a conditional effect would reflect optimal prediction. There were no selected features for CBCL internalizing scores.

We then assessed how individual-level (dis)similarity from the group influenced predictive performance by examining the interaction of ICC and elastic net-predicted score on observed outcome score (Tables 2 and 3).

#### 3.3.1 Hypothesis 2A

**Fig. 4.**
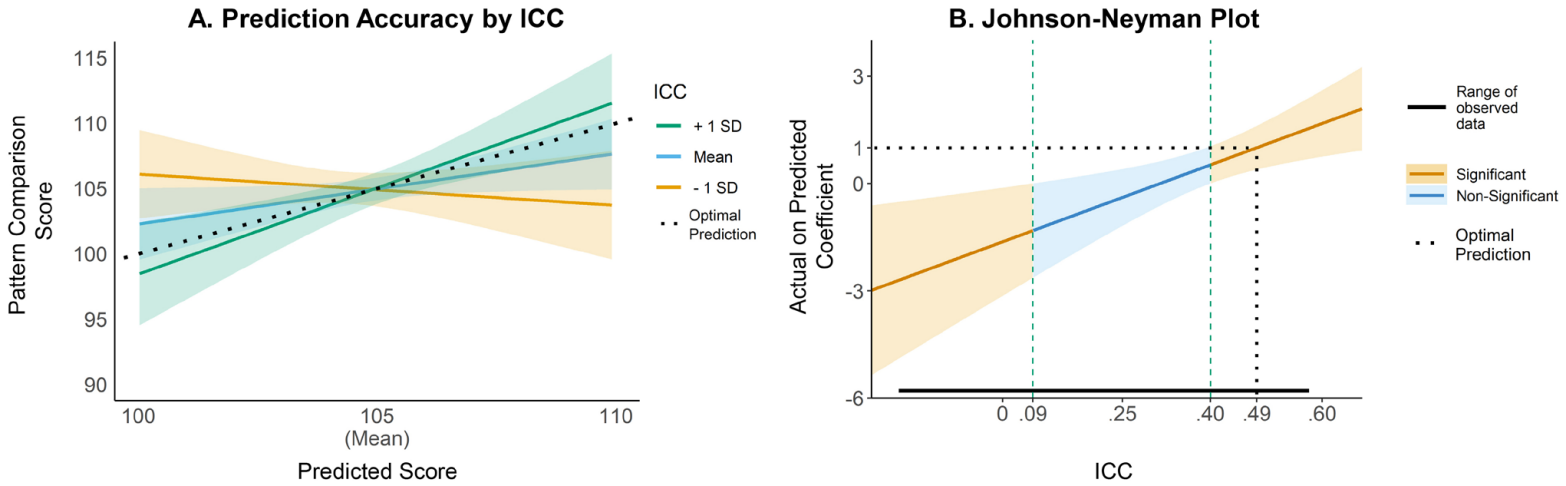
Interaction of predicted pattern comparison score and ICC. *Note*. (A) Differences in predictive performance from ROI-based rsFC by group-to-individual similarity (ICC) in the testing set. A slope of 1 (dotted line) reflects optimal prediction. Prediction of pattern comparisons scores was better (closer to 1) for individuals more similar to the group (higher ICC). Individuals with ICC less than 1 SD from the mean (0.28) had *negative* predictive performance. (B) A Johnson–Neyman plot visualizes the values of ICC that significantly moderate the relationship between predicted scores and observed scores. For individuals with an ICC higher than about 0.40 (near ICC mean of 0.41), prediction was significantly improved and close to 1 for the observed range of data. For individuals with an ICC less than 0.09, prediction was significantly worse.

*Higher ROI-based group similarity (ICC) will be associated with stronger relationships between the predicted and observed outcome values.* Hypothesis 2A was only partially supported. There was one significant interaction with higher ICC scores being associated with better prediction of pattern comparison scores in both the training and testing sets in ROI-based models (Fig. 4). While this finding suggests some preliminary evidence of higher similarity to the group improving individual-level prediction, the ensemble pattern of non-significant interactions does not provide strong support for this hypothesis.

#### 3.3.2 Hypothesis 2B

*Higher network-based group similarity (ICC) will be associated with stronger relationships between the predicted and observed outcome values.* Hypothesis 2B was not supported. No interactions between ICC and predicted scores were significant in network-based models.

### 3.4 Sensitivity analyses

#### 3.4.1 Feature reduction

To examine the impact of noise and number of features on group-to-individual rsFC ICC, we repeated ROI-based analyses using (1) a stricter interindividual variance threshold for FC features and (2) a stricter ROI inclusion threshold of tSnR values 1 standard deviation below the mean. Relative to results using FC features in the top 10% of interindividual variance, selecting the FC features with the top 75% of interindividual variance (4,302 features) increased mean ICCs to 0.49 (SD = 0.14) in the training set and 0.50 (SD = 0.12) in the testing set. When selecting the top 50% of features (8,603 features), mean ICCs further increased to 0.57 (SD = 0.12) in the training set and 0.57 (SD = 0.11) in the testing set. Alternatively, when selecting the FC features with the top 5% of interindividual variance (861 features), mean ICCs decreased to 0.34 (SD = 0.13) in the training set and 0.35 (SD = 0.12) in the testing set. When selecting the top 1% of features (173 features), mean ICCs further decreased to 0.23 (SD = 0.11) in both the training and testing sets. These results suggest that ICC increased with the number of features but remain poor even with a very large number. When using an ROI-inclusion threshold of 1 SD below the mean, the number of features was reduced to 1,370. Results were consistent with the main analyses, with the mean ICC in the training set of 0.40 (SD = 0.14) and in the testing set of 0.41 (SD = 0.13).

#### 3.4.2 Covariates

Age was not significantly associated with ROI-based or network-based ICC in the training (ROI-based: b = -1.71*10-5, SE = 1.04*10-4, p = .87; network-based: b = 2.03*10-5, SE = 6.53*10-5, p = 0.76) or testing (ROI-based: b = 1.97*10-5, SE = 4.50*10-5, p = 0.66; network-based: b = 1.23*10-5, SE = 2.49*10-5, p = 0.62) sets. Sex was not significantly associated with ROI-based or network-based ICC in the training set (ROI-based: b = -0.002, SE = 0.01, p =0.84; network-based: b = -0.01, SE = 0.01, p = 0.22), or network-based ICC in the testing set (b = 0.004, SE = 0.004, p = 0.22), but was significantly associated with ROI-based ICC in the testing set (b = 0.02, SE = 0.01, p = 0.02). There was marginal evidence of an effect of site, with one significant effect. Individuals from site 19 (n = 69 in training and n = 50 in test) had lower ICCs in the training (ROI-based: b = -0.55, SE = 0.05, p < .001; network-based: b = -0.27, SE = 0.04, p < .001) and testing (ROI-based: b = -0.51, SE = 0.04, p < .001; network-based: b = -0.28, SE = 0.03, p < .001) sets. Additionally, higher mean framewise displacement was significantly associated with lower ICC in both the training (ROI-based: b = -0.11, SE = 0.01, p < .001; network-based: b = -0.16, SE = 0.01, p < .001) and testing (ROI-based: b = -0.15, SE = 0.01, p < .001; network-based: b = -0.27, SE = 0.02, p < .001) sets.

We examined the significance of the focal interactions when controlling for sex, site, and framewise displacement with additional moderated regression models. The interaction predicting training and testing set pattern comparison scores in ROI-based models remained significant when controlling for sex, site, and motion (p < .01 for training and testing sets). Additionally, the interaction was significant in network-based CBCL externalizing models when controlling for site (p = .01), but only in the training set. Like for pattern comparison, higher ICC scores were associated with better prediction of CBCL externalizing scores when controlling for site. This interaction was not significant in the main models (p = .055).

## 4 Discussion

Most rsFC research uses between-person study designs that rely on group averages for generalizing inferences. However, precision imaging studies have found that individuals have trait-like differences that are obscured in group averages (Gordon et al., 2017; Gratton et al., 2018; Seitzman et al., 2019). Heterogeneity between persons may limit the ability of group averages to reflect individual-level functioning and predict key outcomes for individuals distinct from the group (Mattoni et al., 2025). This registered report quantified interindividual heterogeneity of rsFC strength in a large population-based adolescent sample and examined how individual dissimilarity from the group impacted behavioral prediction. We found substantial heterogeneity in ROI-based rsFC, with poor reliability between the group-average rsFC model and *every* individual. In contrast, we found evidence of homogeneity of network-based rsFC, with the group-to-individual reliability for the majority of individuals being excellent. Hypotheses that behavioral prediction from rsFC networks would be better for individuals with higher group-to-individual ICC values were mostly not supported, with only one case where ICC significantly moderated prediction. Limited clinical variability was a notable limitation for tests examining how group-to-individual dissimilarity moderated clinical prediction.

Heterogeneity between individuals is an important factor in fMRI research, especially for brain–behavior associations (Feczko et al., 2019; Gell et al., 2024; Gratton et al., 2020). While prior work with intensively scanned samples has found important differences between individuals, observations have been mostly qualitative and based on network topology. Here, we operationalized heterogeneity of rsFC strength as group-to-individual ICC estimates in a large population-based sample. ROI-based rsFC similarity was poor across the entire sample and was not due to head motion (.01 mm difference in head motion predicted .002 difference in ICC), age, or sex. The poor group-to-individual ICC estimates for ROI-based rsFC were widespread, as the group-averaged rsFC model was a poor reflection of every individual in both the training and testing sets. This can be visually observed in Figure 2A, where even the individual with the highest group-to-individual ICC (.60) is still notably distinct from the group. One possible explanation for the substantial heterogeneity may be the unconstrained nature of resting-state scans. However, similar heterogeneity was not observed in network-based resting-state models.

Beyond poor individual-level precision, heterogeneity in ROI-based models also breaks necessary assumptions for ergodicity (Hunter et al., 2024; Mattoni et al., 2025; Molenaar, 2004). Non-ergodicity suggests that the average rsFC network for many individuals observed once (interindividual) cannot be assumed to be equal to average rsFC network of any one individual observed numerous times (intraindividual). Perhaps more importantly, interindividual *variance* in rsFC networks is not necessarily equivalent to intraindividual variance (Fisher et al., 2018). Though not examined here, this non-ergodicity may have critical implications for brain–behavior associations across interindividual and intraindividual levels of analysis. That is, since interindividual and intraindividual variance in rsFC are distinct, brain–behavior associations may also be distinct at interindividual and intraindividual levels (Mattoni et al., 2025). The few intensively sampled rsFC studies support this likelihood. For example, the My Connectome Project found that visual, somatomotor, and dorsal attention networks have high intraindividual variability relative to other networks, but low interindividual variability (Mueller et al., 2013; Poldrack et al., 2015). As an applied clinical example, Lynch et al. (2024) found a robust interindividual association between the salience network and depression, but no significant association at an intraindividual level. Overall, rsFC non-ergodicity suggests that intraindividual inferences cannot be assumed from interindividual study, but must be directly examined from within-person study designs (Mattoni et al., 2025).

In contrast to ROI-based models, network-based rsFC was largely homogeneous across individuals. Importantly, sensitivity analyses suggested that the increase in group-to-individual similarity was not simply due to data aggregation (Fig. 3), and feature reduction *lowered* ICC estimates in ROI-based analyses. Network-based homogeneity is broadly consistent with findings of the generalizability of large-scale networks structures across individuals and species (Miranda-Dominguez et al., 2014; Smith et al., 2013; Yeo et al., 2011). In contrast, precision studies of large-scale network structures consistently find individual-level deviations from the group (Dworetsky et al., 2024; Gordon et al., 2017; Seitzman et al., 2019). Our results do not necessarily conflict with these prior findings of heterogeneity due to two key methodological differences. First, our analytical focus was on connectivity strength with a priori network assignments, rather than differences in network topology (Gordon & Nelson, 2021). Second, our ICC-based operationalization of heterogeneity is focused on overall variance between and within networks rather than specific differences in a single network. Specific differences are still observable between groups and individuals in our analysis (e.g., compare connectivity between the cingulo-parietal and default mode networks in Figure 2B for the maximum ICC individual vs. group average). Moreover, while the vast majority of individuals were well reflected by the group, there are multiple individuals with more moderate group similarity (e.g., -2 SD individual in Fig. 2B) that has more widespread differences. This individual-level deviation from the group suggests that while network models broadly represent most individuals, they do not fully capture important traits of every individual. Furthermore, prior work has shown that group-level rsFC deviation may be behaviorally and clinically meaningful (Dworetsky et al., 2024; Gratton et al., 2020; Seitzman et al., 2019), although ICC was not predictive of outcomes here. Overall, our findings suggest that patterns of resting-state connectivity *strength* within and between prespecified networks are largely consistent across individuals.

Our second goal was to examine how individual-level (dis)similarity from the group impacted prediction of clinical and cognitive outcomes. We hypothesized that, for both ROI-based and network-based rsFC models, prediction would be more accurate for individuals with rsFC more similar to the group. While this hypothesis was supported for the ROI-based prediction of pattern comparison performance, the overall collection of results does not support it. For pattern comparison, a Johnson–Neyman plot (Fig. 4) shows that prediction accuracy was significantly higher for individuals with ICC greater than the sample mean and significantly worse—to such an extent that the relationship between predicted and observed scores was negative—for individuals with an ICC more than 2 standard deviations below the sample mean. Moreover, this interaction was robust, significant in both the training and testing sets, with and without controlling for motion and scanning site. A similar interaction was also found in registered sensitivity analyses for prediction of CBCL externalizing scores. However, this result was not robust, only found when controlling for site in the training set (p = .055 in main registered analysis). Together, these findings provide some preliminary evidence that behavioral prediction is worse for individuals more distinct from the group. However, this interpretation is cautioned by the null results in most ROI-based models, and all network-based models. With the number of tests, it is possible the significant moderation of pattern comparison is a false positive. Alternatively, there are notable limitations in variability that may explain the lack of significant findings for most models. Distributions for clinical outcomes were highly positively skewed, with most individuals near the floor (see Supplementary Data). Additionally, distributions for ICC estimates were narrow, especially for network-based models (Fig. 3). Minimal interindividual variability provides little opportunity to detect interindividual associations, highlighting a key future development of more sensitive psychopathology measures and specific measures of heterogeneity.

Although behavioral prediction outside the context of group similarity was not a primary focus of this study, there are several interesting observations. First, predictive performance was low, with correlations ranging from 0.00 to 0.14 in the testing set. This predictive performance is similar to that found in the baseline sample with similar features and outcomes (Marek et al., 2022). Thus, while rsFC is increasingly examined as a predictor or “biomarker” of clinical outcomes, it has overall low predictive value (though prediction was likely impacted by the little clinical variability in the sample). Second, models were highly overfit to the training set, especially in ROI-based models, with predictive performance reaching as high as r = .48. Consistent model overfit highlights the challenges of generalizable prediction and the necessity of external tests, especially for high-dimensional models. Third, ROI-based and network-based models had variable predictive performance across measures, suggesting that neither is systematically better, and each may be used for different purposes. Fourth, we performed feature selection based on theoretical relevance of functional networks, higher intraindividual signal-to-noise ratios, and higher interindividual variance. With a reduced features set, our ROI-based models performed similarly to other ABCD studies using the full rsFC matrix (e.g., Chen et al., 2022, 2023; Marek et al., 2022). Future studies may consider similar feature reduction strategies for more interpretable models (i.e., fewer features, more theoretical relevance, less noise).

This study had several strengths, including a large sample and prediction in external data, a registered report format to reduce researcher bias, and the quantification of heterogeneity and impacts on prediction. Several limitations should also be considered. First, we used intraclass correlations to examine the reliability between a group-average rsFC model and each individual. While this operationalization reasonably captures individual differences from the group (Fig. 2), ICC is a ratio of between-group and total variance. Thus, as used here, ICC is sensitive to variance across connectivity measures within individuals and is not an absolute measure of dissimilarity. Future development of absolute measures of heterogeneity is critical to this growing area of interest. Second, there was limited between-person variance both for behavioral outcomes and ICC estimates, especially for network-based approaches. Limited variance may have hindered sensitivity to test the focal interactions. Furthermore, psychopathology outcomes in ABCD are heavily positively skewed. Although elastic net models do not have normality assumptions, skewness can limit interpretability and predictive accuracy. Third, while the sample size was relatively large, functional connectivity strength has modest retest reliability (Noble et al., 2019), limiting statistical power. Fourth, our motion-based exclusion criteria skewed the sample to have relatively more White, wealthy, and female participants, reducing generalizability. Future studies should leverage methodological developments that preserve higher motion data to increase representativeness of population-based analyses (Ramduny et al., 2025). ROI-based exclusion criteria also removed all subcortical regions, which may be of key importance to clinical outcomes (Lecciso & Colombo, 2019; Northoff et al., 2011). Finally, there is substantial developmental variability in the ABCD sample that may contribute to functional connectivity heterogeneity. Future work should also empirically assess heterogeneity in other large samples across development.

### 4.1 Conclusions

This registered report examined the heterogeneity of resting-state networks in the Adolescent Brain Cognitive Development study. A group-averaged region-based functional connectivity network poorly reflected each individual in the sample. Robust heterogeneity suggests non-ergodicity of resting-state networks, such that intraindividual inferences are unsupported from the interindividual study. Additionally, while there was minor evidence that heterogeneity directly limited prediction of behavioral outcomes, most hypotheses were not supported. Predictive models were hindered by little variance in heterogeneity estimates and behavioral outcomes. In contrast, large-scale network-based connectivity models were well represented by the group. Results suggest that within-network and between-network connectivity strength is largely similar across individuals. Overall, this study raises concerns about the use of group-averaged region-based connectivity models for individual-level inferences. Further attention to intraindividual study designs and consequences of heterogeneity is necessary.

## Supplementary Material

Supplementary Material

## Data Availability

Data from the Adolescent Brain Cognitive Development (ABCD) Study are available with a DUC from https://www.nbdc-datahub.org/. Data from the ABCD Community Collection (3165) can be found at https://nda.nih.gov/edit_collection.html?id=3165. Code to reproduce results can be found at https://osf.io/kdx65.

## Footnotes

* Deviation 1: The stage 1 report identified “nihtbx_flanker_rawscore” and “nih_pattern_rawscore” as the designated variables. Upon accessing the data, it became evident these variables were not fully scored for speed and accuracy and were inappropriate for analysis. We instead used uncorrected scores, similar to previous ABCD analyses (e.g., Marek et al., 2022).

† Deviation 2: The stage 1 report incorrectly indicated that tSnR would be calculated in the whole sample for exclusions, rather than the training set. Using the whole sample would inappropriately break independence of training and testing sets (Scheinost et al., 2019), so ROI feature selection was completed in the training set independently and subsequently applied to the testing set. However, sensitivity analyses revealed that an identical set of ROIs would be chosen with either approach.

‡ Deviation 3: The stage 1 report incorrectly stated that ICC necessarily increases with number of features. While we still present ROI-based ICCs, ROI-based ICCs with a reduced feature set (see sensitivity analyses), and network-based ICCs, we consider all results separately rather than prioritizing network-based ICCs as stated in stage 1.
