# Supplementary material for: Functional connectivity heterogeneity and consequences for clinical and cognitive prediction: Stage 2 registered report
Source: Imaging Neurosci (Camb). 2025 Aug 12;3:IMAG.a.107. doi: 10.1162/IMAG.a.107 (PMC12344514; doi:10.1162/IMAG.a.107)

Table S1. Sample Demographics

	Category	Baseline Sample (N = 11,878)	2-Year Train Sample (N = 1165)	2-Year Test Sample (N = 1165)
Age in years	*Mean (SD)*	9.91 (0.63)	12.02 (0.67)	12.05 (0.66)
Sex	Male *(%)*	48.41	45.92	47.64
	Female *(%)*	51.59	54.08	52.36
Race/Ethnicity	White *(%)*	53.30	63.09	61.37
	Black *(%)*	13.96	10.21	9.44
	Hispanic *(%)*	21.68	15.88	17.60
	Asian *(%)*	2.43	1.37	1.80
	Other *(%)*	9.62	9.44	9.79
Family Income ($)	<25K *(%)*	12.35	7.38	7.30
	25K-49.999K *(%)*	12.63	13.30	12.79
	50K-74.999K *(%)*	12.19	14.25	14.85
	75K-99.999K *(%)*	13.33	15.28	16.91
	100K-199.999K *(%)*	29.02	31.16	30.13
	200K + *(%)*	11.85	11.59	12.10
Highest Parental Education	<GED *(%)*	6.72	4.29	4.03
	GED *(%)*	26.18	23.35	22.83
	2-year degree *(%)*	12.17	12.79	12.96
	Bachelor's *(%)*	28.46	31.76	34.51
	Graduate *(%)*	26.38	27.73	25.67
CBCL	Internalizing *Mean (SD)*	5.05 (5.53)	4.61 (5.41)	4.83 (5.51)
	Externalizing *Mean (SD)*	4.46 (5.87)	3.36 (4.81)	3.58 (4.86)
BPM*	Internalizing *Mean (SD)*	1.82 (2.09)	1.62 (2.05)	1.76 (2.22)
	Externalizing *Mean (SD)*	1.95 (2.00)	1.95 (1.86)	2.06 (2.01)
Neurocognition	Flanker Test *Mean (SD)*	93.00 (9.15)	101.01 (7.01)	100.99 (6.96)
	Pattern Comparison Test *Mean (SD)*	88.06 (14.59)	105.36 (14.81)	105.22 (14.09)
Framewise Displacement	*Mean (SD)*	0.32 (0.39)	0.08 (0.04)	0.08 (0.05)

Note. CBCL = Childhood Behaviroal Checklist. BPM = Brief Problem Monitor. *Baseline youth-reported psychopathology is from 6-month-follow-up because the Brief Problem Monitor was not administered at baseline.

Figure S1. Overview of Analytic Plan

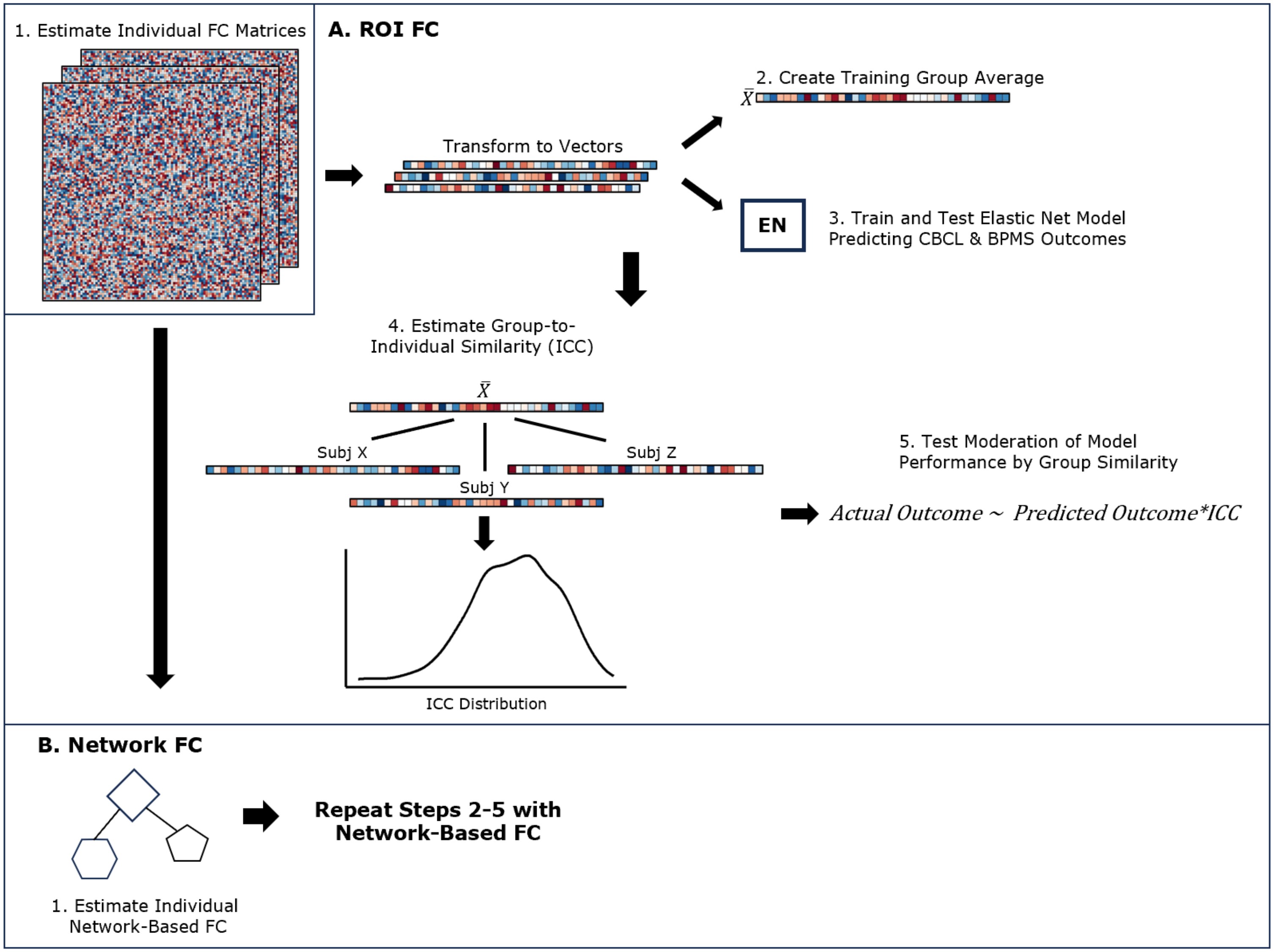

Figure 2. All steps will be repeated using **(A)** ROI-based rsFC and **(B)** network-based rsFC. The only practical difference occurs in step 1. Step 1: Estimate rsFC matrices for each individual, transform lower triangle of each matrix into a vector. Step 2: In the training sample, average across all participants to create a group average rsFC network. Step 3: Using the training subset, train an elastic net model that predicts each of the four clinical outcomes from rsFC matrices. Use final model to estimate predicted outcomes for all individuals in train and test sets. Step 4: Estimate the intraclass correlation coefficient (ICC) value between the training group average and each individual in the training and test subsets. Examine distributions separately **(Research Question 1)**. Step 5: Test moderation of model performance by group similarity **(Research Question 2)**. Model performance is the main effect of actual outcome by predicted outcome and group similarity is rsFC group-individual ICC. The interaction effect is the focus of the study.

Figure S2. Distributions of Outcomes in the Testing Sample

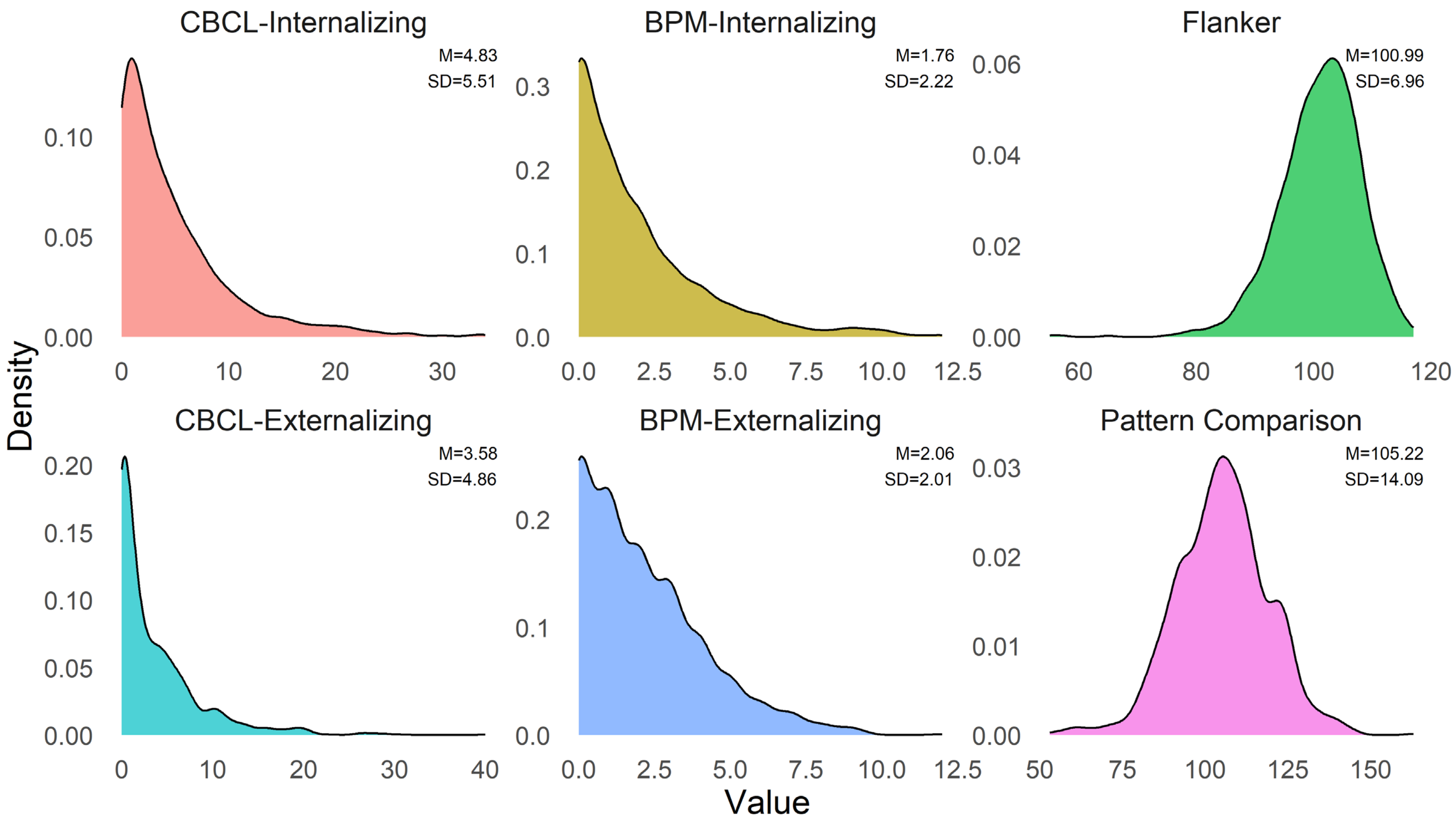

Figure S3. Relationship between ICC and Outcomes in the Testing Sample

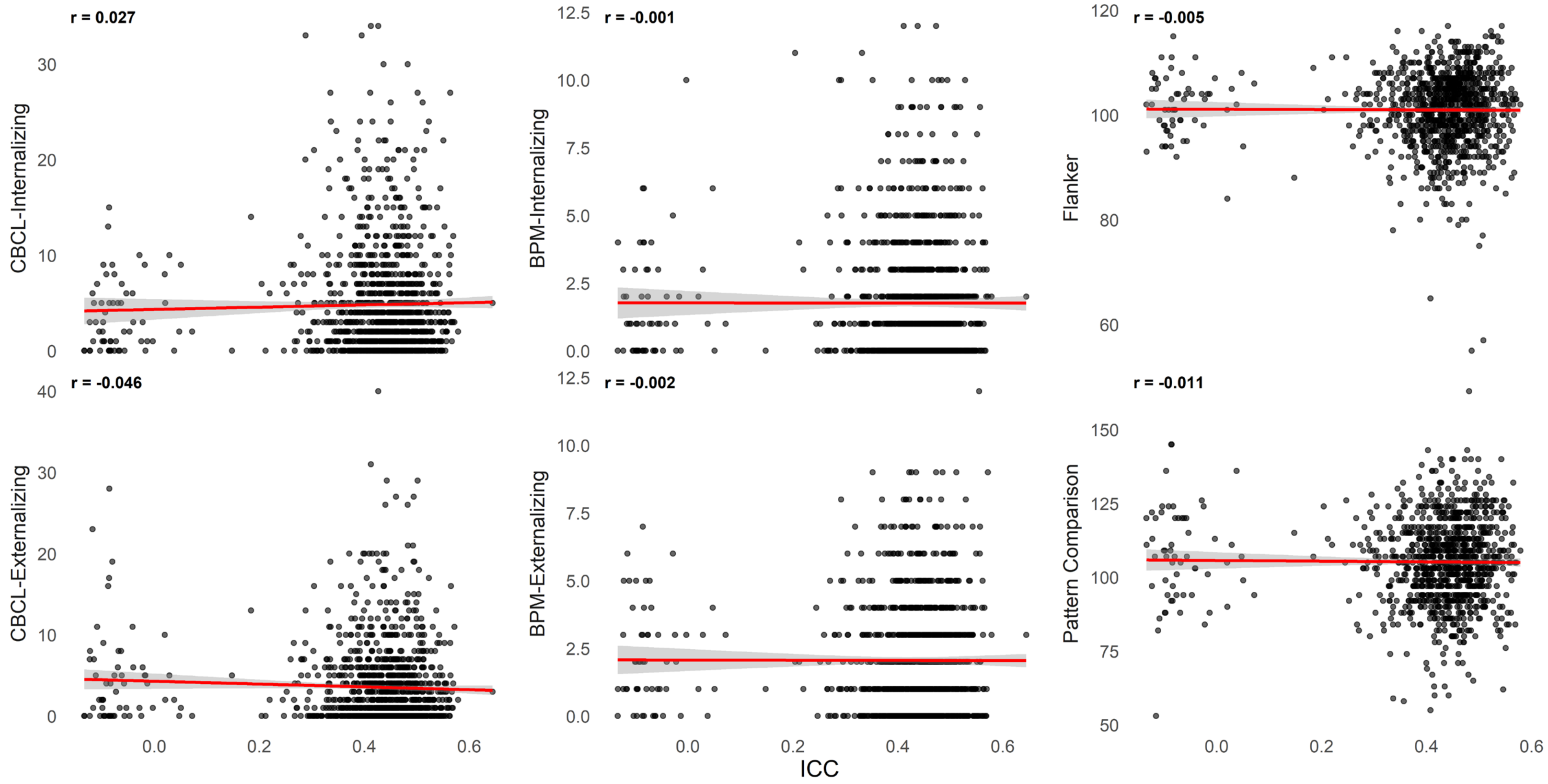

Figure S4. Relationship between Predicted and Observed Scores in the Testing Sample

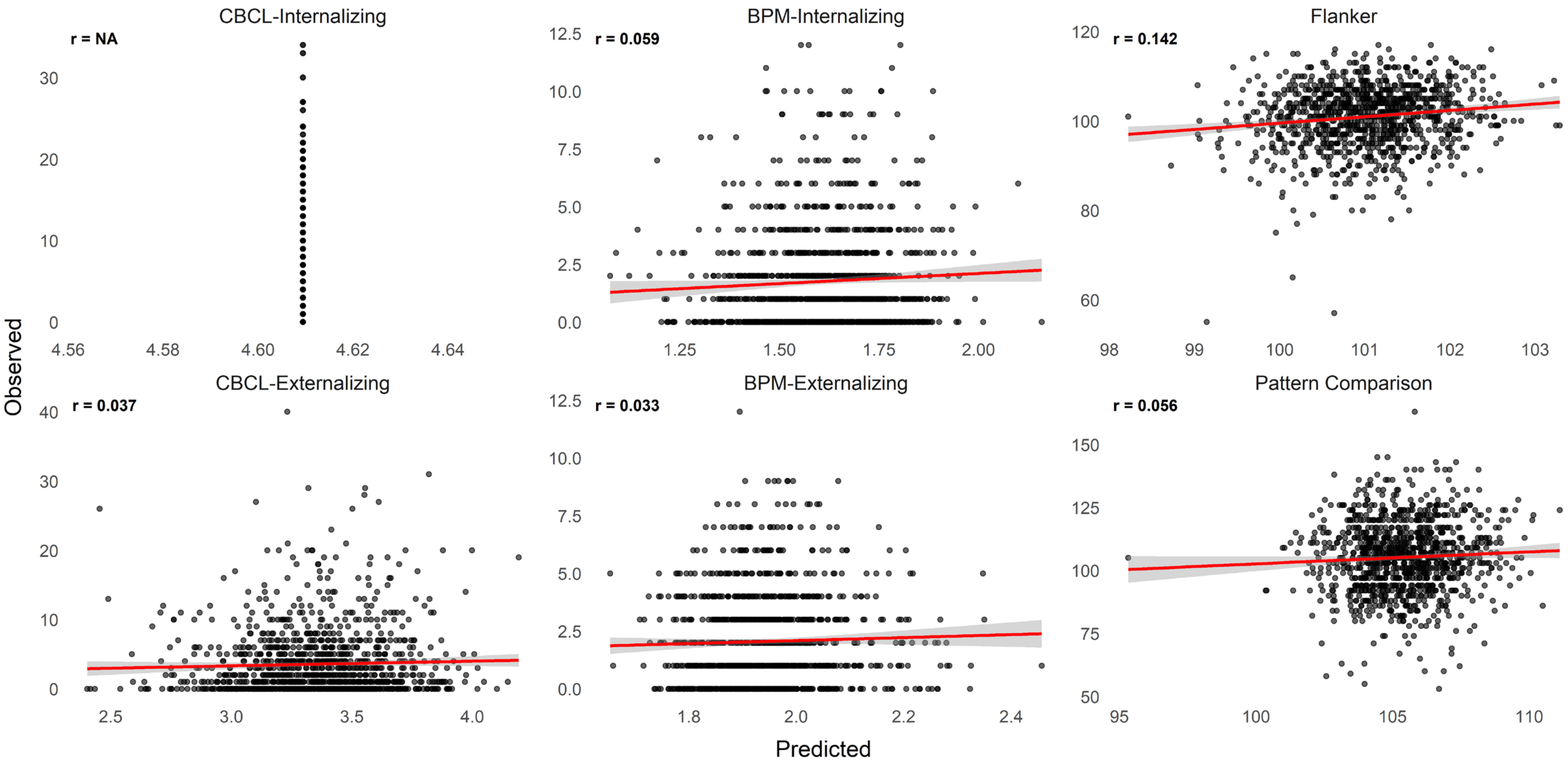

Supplement: Supplementary Material [file IMAG.a.107_supp.pdf]
